# Supplementary material for: Genetic Drift Dominates Genome-Wide Regulatory Evolution Following an Ancient Whole-Genome Duplication in Atlantic Salmon
Source: Genome Biol Evol. 2021 Mar 22;13(5):evab059. doi: 10.1093/gbe/evab059 (PMC8140206; doi:10.1093/gbe/evab059)
Supplement: evab059_Supplementary_Data [file evab059_supplementary_data.docx]

**Supplementary Figures**

**Figure S1.** Number of expressed genes (RPKM > 1) in 14 salmon tissues from (Lien et al. 2016).


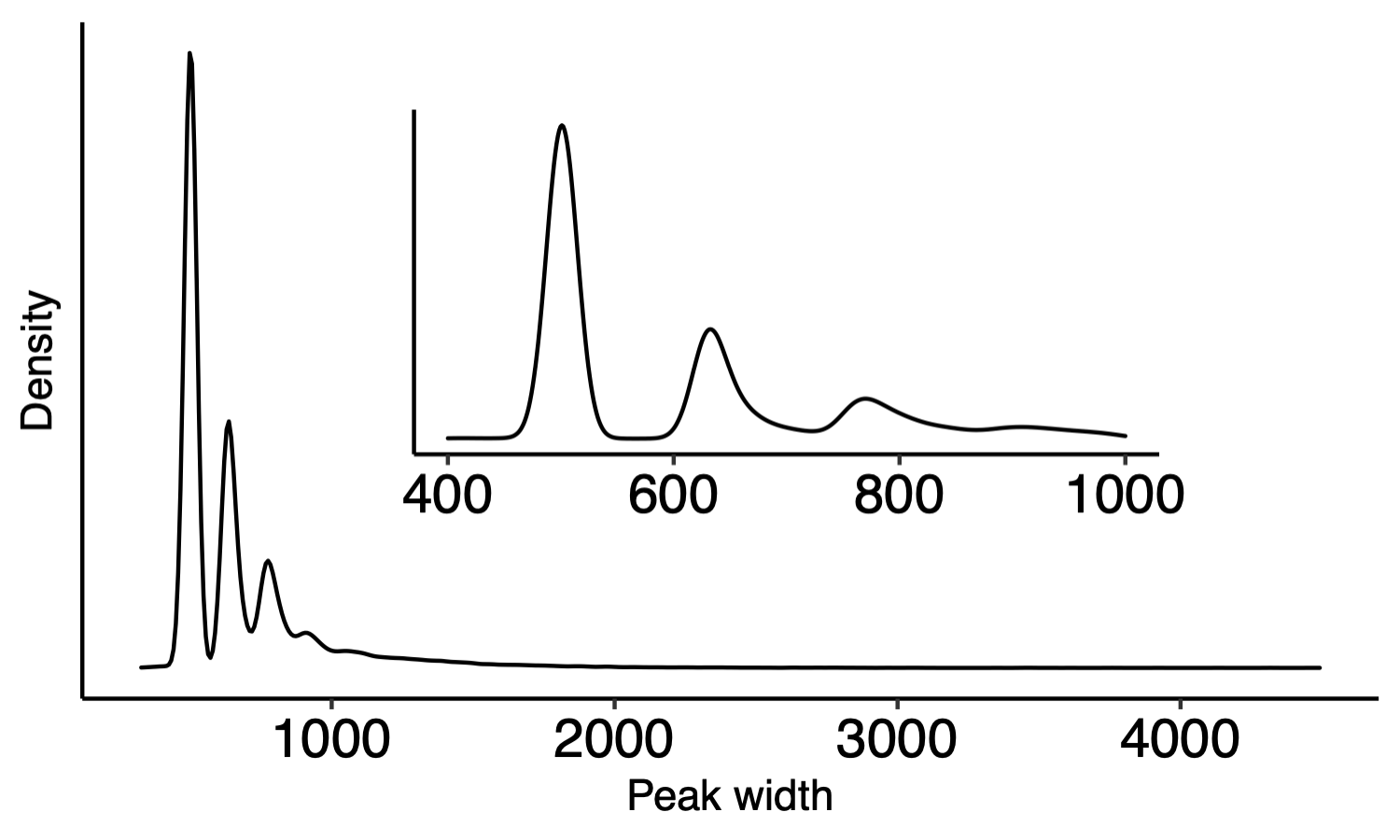


**Figure S2.** Distribution of H3K27ac peak widths identified using *HOMER*. The default peak width of 500 bp was used. Distribution of peak widths shows a periodical pattern that is consistent with peaks representing regions covering different numbers of acetylated nucleosomes.

**Figure S3.** Distance between proximal peaks and closest TSS.


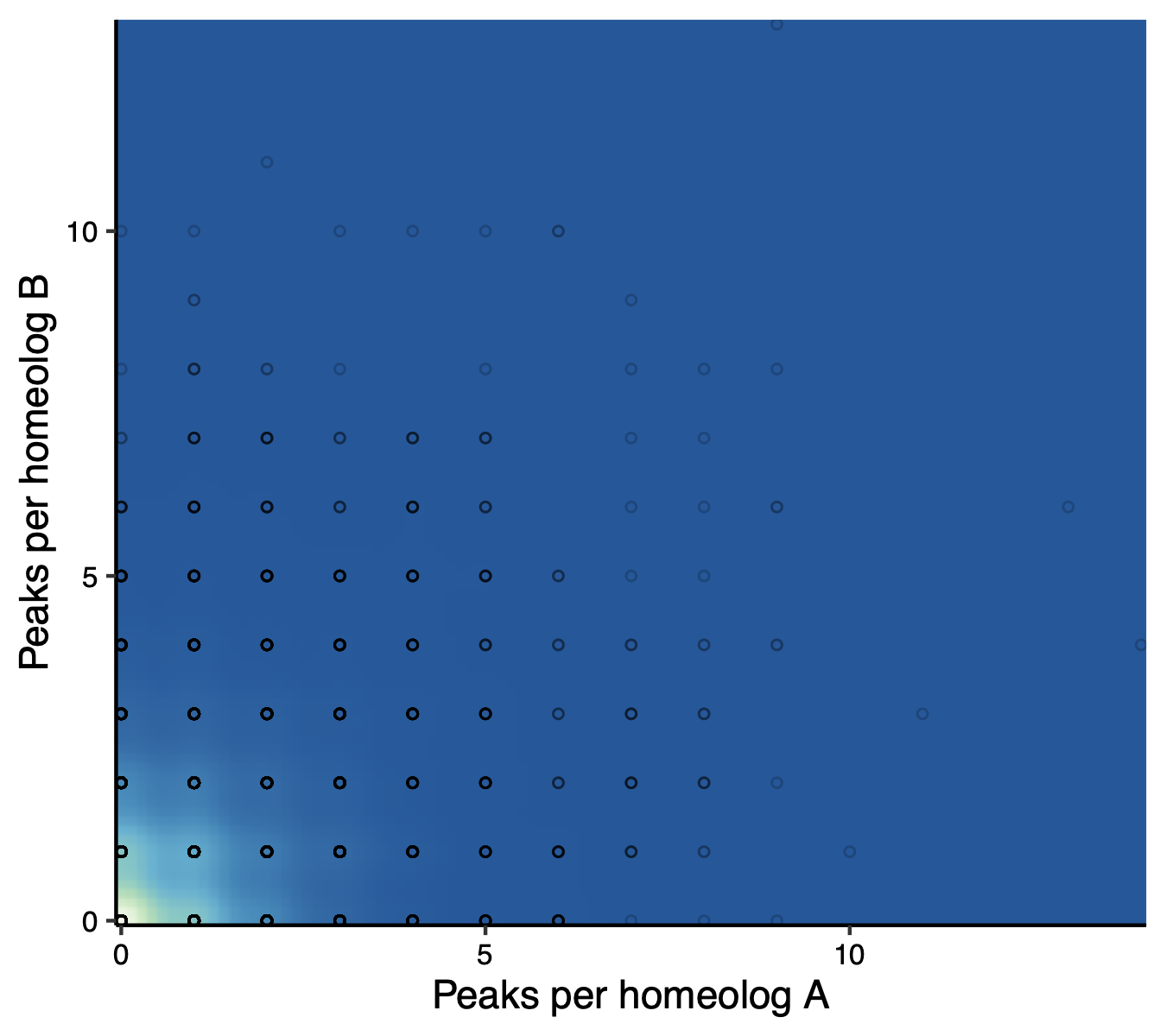


**Figure S4.** The number of H3K27ac peaks assigned to ohnologs. Background is coloured according to the density of datapoints.

**Figure S5.** Genomic distribution of H3K27ac peaks in (**A**) top ten most diverged homeoblocks and in (**B**) all homeoblocks. Circos plot showing tracks (from outside) for the number of H3K27ac peaks per 1 Mb windows, peak density per 100 Kb for homeoblocks, and links identifying homeoblocks. Links are colored according to residuals from expected 1:1 peak density between homeoblocks (darker color indicates stronger differences in peak density, similar to Fig 3B).

**Figure S6.** Density of genes versus density of H3K27ac peaks per 1Mb windows.

**Table S1** ChIPmentation sequencing samples. *Fastp filters: --low_complexity_filter, ** Bowtie2 parameters: --very-sensitive --maxins 1500 --end-to-end, ** samtools filters: -F 256 -q 20

**Table S2** Accession ID, sample ID and mean and median sequencing coverage for each sample in the re-sequencing dataset. Measured after mapping the raw reads from Barson et al. (2015) to the Atlantic salmon reference genome and removing PCR duplicates, prior to variant calling.

| **run accession** | **sample** | **mean coverage** | **median coverage** |
| --- | --- | --- | --- |
| ERR1013407 | Alta_12_0001 | 7.520952 | 8 |
| ERR1013495 | Alta_12_0124 | 3.349194 | 3 |
| ERR1013583 | Alta_12_0228 | 7.705269 | 8 |
| ERR1013463 | Arga_12_0075 | 7.728542 | 8 |
| ERR1013471 | Arga_12_0082 | 9.305405 | 10 |
| ERR1013479 | Arga_12_0089 | 13.245793 | 14 |
| ERR1013647 | Jols_13_0001 | 10.631584 | 11 |
| ERR1013639 | Jols_13_0009 | 8.723167 | 9 |
| ERR1013655 | Jols_13_0027 | 7.64258 | 8 |
| ERR1013447 | Nams_12_0024 | 10.828244 | 11 |
| ERR1013455 | Nams_12_0071 | 10.654392 | 11 |
| ERR1013439 | Nams_12_0201 | 6.65685 | 7 |
| ERR1013423 | Naus_12_0014 | 9.373985 | 10 |
| ERR1013415 | Naus_12_0037 | 7.585018 | 8 |
| ERR1013431 | Naus_12_0059 | 12.050208 | 13 |
| ERR1013615 | Repp_12_0007 | 8.360628 | 9 |
| ERR1013623 | Repp_12_0023 | 6.412691 | 6 |
| ERR1013631 | Repp_12_0034 | 12.499478 | 13 |
| ERR1013487 | Uts_11_15 | 7.155256 | 7 |
| ERR1013511 | Uts_11_17 | 19.169955 | 21 |
| ERR1013519 | Uts_11_24 | 9.167601 | 10 |
| ERR1013527 | Uts_11_26 | 5.419665 | 5 |
| ERR1013535 | Uts_11_27 | 6.556109 | 7 |
| ERR1013543 | Uts_11_28 | 6.834338 | 7 |
| ERR1013551 | Uts_11_29 | 4.531277 | 4 |
| ERR1013559 | Uts_11_30 | 6.543761 | 7 |
| ERR1013567 | Uts_11_31 | 6.588743 | 7 |
| ERR1013575 | Uts_11_39 | 2.994131 | 3 |
| ERR1013591 | Uts_11_46 | 7.094451 | 7 |
| ERR1013599 | Uts_11_52 | 3.360987 | 3 |
| ERR1013607 | Uts_11_53 | 8.927331 | 9 |

**Table S3** Reference versions and ftp links for genomes used in the multispecies whole genome alignment. Reference species in bold (brown trout)

| Common name | Scientific name | Version | Available from |
| --- | --- | --- | --- |
| Arctic charr | *Salvelinus alpinus* | ASM291031v2 | <ftp://ftp.ncbi.nlm.nih.gov/genomes/all/GCF/002/910/315/GCF_002910315.2_ASM291031v2/GCF_002910315.2_ASM291031v2_genomic.fna.gz> |
| Atlantic salmon | *Salmo salar* | ICSASG_v2 | <ftp://ftp.ncbi.nlm.nih.gov/genomes/all/GCF/000/233/375/GCF_000233375.1_ICSASG_v2/GCF_000233375.1_ICSASG_v2_genomic.fna.gz> |
| brown trout | *Salmo trutta* | fSalTru1.1 | <ftp://ftp.ncbi.nlm.nih.gov/genomes/all/GCF/901/001/165/GCF_901001165.1_fSalTru1.1/GCF_901001165.1_fSalTru1.1_genomic.fna.gz> |
| coho salmon | *Oncorhynchus kisutch* | Okis_V1 | <ftp://ftp.ncbi.nlm.nih.gov/genomes/all/GCF/002/021/735/GCF_002021735.1_Okis_V1/GCF_002021735.1_Okis_V1_genomic.fna.gz> |
| grayling | *Thymallus thymallus* | ASM434828v1 | <ftp://ftp.ncbi.nlm.nih.gov/genomes/all/GCA/004/348/285/GCA_004348285.1_ASM434828v1/GCA_004348285.1_ASM434828v1_genomic.fna.gz> |
| huchen | *Hucho hucho* | ASM331708v1 | <ftp://ftp.ncbi.nlm.nih.gov/genomes/all/GCA/003/317/085/GCA_003317085.1_ASM331708v1/GCA_003317085.1_ASM331708v1_genomic.fna.gz> |
| northern pike | *Esox lucius* | Eluc_v4 | <ftp://ftp.ncbi.nlm.nih.gov/genomes/all/GCF/004/634/155/GCF_004634155.1_Eluc_v4/GCF_004634155.1_Eluc_v4_genomic.fna.gz> |
| rainbow trout | *Oncorhynchus mykiss* | Omyk_1.0 | <ftp://ftp.ncbi.nlm.nih.gov/genomes/all/GCF/002/163/495/GCF_002163495.1_Omyk_1.0/GCF_002163495.1_Omyk_1.0_genomic.fna.gz> |
| sockeye salmon | *Oncorhynchus nerka* | Oner_1.0 | <ftp://ftp.ncbi.nlm.nih.gov/genomes/all/GCF/006/149/115/GCF_006149115.1_Oner_1.0/GCF_006149115.1_Oner_1.0_genomic.fna.gz> |

**Supplementary Analyses**

We used alternative parametrization of *HOMER* to H3K27ac peaks that were suitable for transcription factor motif finding (fixed 500 bp peaks centered on nucleosome free regions). This analysis identified 26,507 replicated peaks, of which 83% were overlapping peaks identified in the main analysis. We additionally identified TF motifs in the original peak set, which gave congruent results. Replicated peaks were over-represented in motifs for general promoter features (TATA-box 28.4% of peaks, *p=*1e-196), as well as for transcription factors implicated in the development of spermatogonia (OCT6 10.1% of peaks, *p=*1e-294), Sertoli and Leydig cells (SMAD4 49.3% of peaks *p=*1e-90, SMAD2 47.3% of peaks *p=*1e-51, SF1 12.2% of peaks *p=*1e-132 and FoxL2 41.9% of peaks *p=*1e-75).

We defined a set of one-to-one ohnologs using an alternative approach as implemented in the software *OrthoFinder* (Emms and Kelly 2015) to test whether the results were robust to analysis strategies. We used northern pike (*Esox lucius*) and rainbow trout (*Oncorhynchus mykiss*) as outgroups in the analysis following *OrthoFinder* basic procedure. Results using this alternative ohnolog set did not differ from the original analysis.
